# Brain regions involved in fractional amplitude of low-frequency fluctuation in cluster headache patients: a resting-state functional MRI study

**DOI:** 10.1186/s12883-022-02863-3

**Published:** 2022-09-07

**Authors:** Yun Chen, Xinbo Xing, Wei Dai, Lixia Tian, Zhao Dong, Shengyuan Yu

**Affiliations:** 1 Department of Neurology, Chinese PLA General Hospital; International headache center, Chinese PLA General Hospital, Beijing, China; 2 Chinese PLA Medical School, Beijing, 100853 China; 3 Department of Neurology, Peking University Shougang Hospital, Beijing, 100144 China; 4 Department of Radiology, Fourth Medical Center of Chinese PLA General Hospital, Beijing, 100048 China; 5 School of Computer and Information Technology, Beijing Jiaotong University, Beijing, 100044 China

**Keywords:** Cluster headache, Resting-state functional magnetic resonance imaging, Fractional amplitude of low-frequency fluctuation, Brain activity

## Abstract

**Background:**

We used resting-state functional magnetic resonance imaging (RS-fMRI) to assess the possible pathogenic role of fALFF in CH. A limited number of studies have reported on fractional amplitude of low-frequency fluctuation (fALFF) in cluster headache (CH).

**Methods:**

RS-fMRI scans of 23 patients with CH were obtained (11with left-sided headache and 12 with right-sided headache), along with scans of 23 age- and sex-matched normal controls. The RS-fMRI data were analyzed to explore abnormal brain activity in the left CH and right CH patients during the non-painful state in one cluster period. fALFF was compared between patients and controls, and correlation analysis between the regional mean fALFF values and clinical characteristics was performed.

**Results:**

A decrease in fALFF was detected in the left cerebellum, left lentiform nucleus, left frontal lobe, left anterior cingulate, and right postcentral gyrus in the left CH group compared to the controls, while a decrease of fALFF was detected in the right cerebellum, right cingulate gyrus, right superior parietal lobule, right inferior parietal lobule, right postcentral gyrus, and left precuneus in the right CH group. No patient had a region with increased fALFF. A moderate correlation was observed between some regional mean fALFF values and the clinical characteristics.

**Conclusions:**

We deduced that dysfunction in multiple brain areas is involved in the non-painful state of CH during a cluster period.

**Supplementary Information:**

The online version contains supplementary material available at 10.1186/s12883-022-02863-3.

## Introduction

Cluster headache (CH) is a male-predominant [1], excruciating and strictly one-sided pain syndrome characterized by attacks accompanied by marked ipsilateral cranial autonomic symptoms, such as lacrimation and conjunctival injection [2]. CH affects about 0.12% of the population [3]. Single attacks last 15–180 min and the frequency of attacks ranges from once every day to eight times per day. CH is considered a chronobiological disorder, where seasonal and circadian rhythmicity affect the likelihood of an attack [4]. Approximately 80–90% of CH patients suffer from episodic headaches [5]. Reference to previous studies, CH is activated by the posterior hypothalamus [6] and regulated by the trigemino-parasympathetic reflex [7]. Several intracranial cortical brain regions are also involved, but the pathogenesis of CH remains unclear [8]. Functional neuroimaging of headache patients has enriched our understanding of the pathophysiology of CH and provided unique insight into this syndrome [9].

As a resting-state functional magnetic resonance imaging (RS-fMRI) signal, fractional amplitude of low-frequency fluctuation (fALFF), which covers the low-frequency power spectrum (0.01–0.08 Hz), reflects the intensity of local spontaneous activity in brain areas by quantifying the low-frequency oscillations (LFOs) [10, 11].fALFF is minimally affected by the cerebrospinal fluid, veins, and physiological noise, and improves the cortical activity detection rate. fALFF is sensitive and specific to spontaneous neuronal activity of the resting brain [12]. fALFF is a powerful marker of group differences in spontaneous brain activity, and brain areas with increased fALFF are associated with the default mode network (DMN) in the resting state [13, 14]. fALFF is now considered reliable for measuring regional spontaneous activity, and for exploring the pathophysiology of neuropsychiatric disorders including Alzheimer’s disease [15], post-stroke depression [16], anxiety, depression [17], and migraine [18, 19].

However, there was no study to detect spontaneous brain activity in patients with CH using fALFF during the resting state. We performed RS fMRI in the non-painful phase of CH in a cluster period, calculated fALFF values, and compared brain activity of left or right CH patients to normal controls, respectively.

## Methods

### Participants

All procedures were approved by the Chinese Ministry of Health and the Ethics Committee of the Chinese PLA General hospital, Beijing, china and were conducted according to the ethical principles of the Declaration of Helsinki. Participants admitted to the International Headache Center of PLA General Hospital between January 2017 and January 2018 were enrolled in the study after providing written informed consent.

The inclusion criteria were as follows: met the diagnostic criteria for CH of the International Classification of Headache Disorders 3rd Edition (ICHD-3) [2]; severe unilateral headache; aged 20–60 years; right-handed; no chronic diseases, including diabetes, hypertension, hypercholesterolemia, cardiovascular and cerebrovascular diseases, or tumors, epilepsy, infectious diseases, connective tissue diseases, other types of chronic pain, or severe anxiety and depression; and no history of alcohol, and other substance abuse. Demographics and the following clinical variables were acquired through interviewing of the patients and review of medical charts: the gender, age, age at onset, duration of headache attack, number of attacks per day, cluster bout duration, disease duration, degree of pain at onset, or accompanying symptoms including nausea, vomiting, photophobia, phonophobia, conjunctive ingestion, lacrimation, nasal congestion, and rhinorrhea. Headache pain degree was evaluated using a visual analog scale (VAS). The control group was healthy people recruited from the staff of the PLA General Hospital and their relatives. The normal controls (NC) were age- and gender-matched with the CH patients and had no history of primary headache, any other type of headache in the past month and no chronic diseases mentioned above.

### RS-fMRI data collection

All subjects were advised to keep their eyes closed, but to remain awake and think of nothing in particular during the scan. The scans were taken with the Sigma 3.0 T magnetic resonance imaging system (Siemens, Munich, Germany), and the functional images were acquired using an echo plane pulse sequence. The scanning parameters were as follows: repetition time (TR) = 2000 ms, echo time (TE) = 30 ms, layer thickness = 3.0 mm, interval = 0.6 mm, matrix = 64 × 64, field of view = 200 × 200 mm, flip angle = 90°, scan duration = 4 min 14 s. There were 33 layers, from the foramen magnum to the top of the head (240 time points and 7920 image frames). Subsequently, a sagittal 3D Tl-weighted image of the whole brain was acquired in 128 layers (TR = 2500 ms, TE = 3.5 ms, layer thickness = 1.0 mm, interval = 0.6 mm, matrix = 256 × 256, field of view = 256 × 256 mm, flip angle = 8°).

### Image data preprocessing

MATLAB 2013b software (MathWorks, Natick, MA, USA) was used for data processing, and the fMRI data were processed using DPARSF software (http://www.rfmri.org). The fMRI data preprocessing method employed herein is well-established [17] and includes eight steps. First, DICOM format data were converted into the NIFTI format (Neuroimaging Informatics Technology Initiative). Second, the first 10 time points were removed to reduce the effect of maladjustments at the beginning of the scan. Then, data from all layers were calibrated to a specific time point using a time correction process, and the functional time series was realigned to correct for head motion across the time series. The brain images of each subject were then transferred to standard space for normalization, to reduce differences between individuals for the next step, i.e., smoothing, in which the effect of spatial noise and differences in brain structure between subjects was reduced using a 4 × 4 × 4 mm smoothing kernel. Finally, after detrending, the low-frequency band wave was used to remove the effect of high frequency signals from respiratory heartbeats, and high frequency noise, to obtain the low-frequency (0.01–0.10 Hz) fluctuations in the resting brain (filtering). After calculating the fALFF value, the Gaussian kernel function was applied for spatial smoothing, and a standardized fALFF diagram was acquired of each participant over the range of 0.01–0.08 Hz. Then, Statistical Parametric Mapping 8 (SPM8) (http://www.fil.ion.ucl.ac.uk/spm) and RESTplus V1.21 (http://www.restfmri.net/forum/REST) software were used for statistical analyses of the fALFF data, and to integrate the fALFF value with the image and determine changes in local brain function. The abnormal brain area of ​​CH patients was defined as the mask, and RESTplus was used to extract the corresponding index values from the center of the region of interest (radius = 6 mm), to derive the mean fALFF value.

### Statistical analysis

Student *t*-test was used to compare difference of VAS between the left CH and right CH patients. The chi-square test or Fisher’s exact test was used to analyze the demographic and other clinical characteristics of the left and right CH patients. Statistical analyses were performed using the SPSS 18.0 software, and *P*-values of<0.05 were considered to be significant. Independent sample tests were performed using Matlab (R2013b) to compare fALFF values of the left or right CH patients to the controls, respectively. RESTplus software was used to extract the corresponding index values. A *P-*value < 0.01 for a minimum volume of 1458 mm^3^ was taken to indicate a significant difference. Pearson’s correlation analysis was performed to assess the relationship between the mean fALFF value across all voxels (in brain regions with abnormal fALFF values) and clinical characteristics of the left or right CH patients.

## Results

### Demographic characteristics

Forty-six volunteers were enrolled in the study, including 23 CH patients and 23 healthy sex- and age-matched controls (supplementary Table 1). For cluster headache group, there were more males in both the left and right CH groups (81.8 and 75.0%). The average age and age at onset of the patients in CH groups were 33.5 ± 10.8 years and 25.0 ± 8.7 years respectively.

### The characteristics and accompanying symptoms of CH

In our patients, the majority of the attacks lasted more than 30 minutes, had a daily frequency of only one attack. Seventy percent of patients experienced severe pain (VAS score > 8). Patients who had a duration of disease more than 10 years and had a duration of the bout longer than 4 weeks, both accounted for 47.8% of the total cases (supplementary Table 2). However, these clinical features did not differ between the left CH and right CH groups.

When CH attacks, the vast majority of patients are accompanied by conjunctival hyperemia (91.3%) and lacrimation (91.3%). In addition, our patients also have nasal congestion (52.2%), rhinorrhea (60.9%), nausea (60.9%), vomiting (39.1%), photophobia (34.8%) and phonophobia (30.4%). No significant differences were observed about the accompanying symptoms between the left and right CH groups (supplementary Table 3).

### fALFF results

**Fig. 1:**
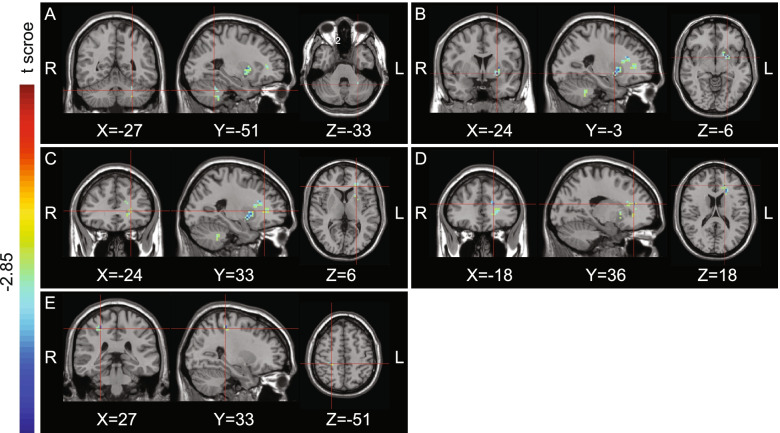
Regions showing significant differences in fALFF between L-CH patients during the non-painful period and normal controls, with a threshold of *P* < 0.01. The color bar indicates the *t*-score of brain regions with fALFF differences between CH patients during the non-painful period and normal controls, R: right; L: left. **A** The left cerebellum (x = − 27, y = − 51, z = − 33). **B** The left lentiform nucleus (x = − 24, y = 3, z = − 6). **C** The left frontal lobe (x = − 24, y = 33, z = 6). **D** The left anterior cingulate(x = − 18, y = 36, z = 18). **E** The right postcentral gyrus (x = 27 y = − 33 z = 51)

**Fig. 2:**
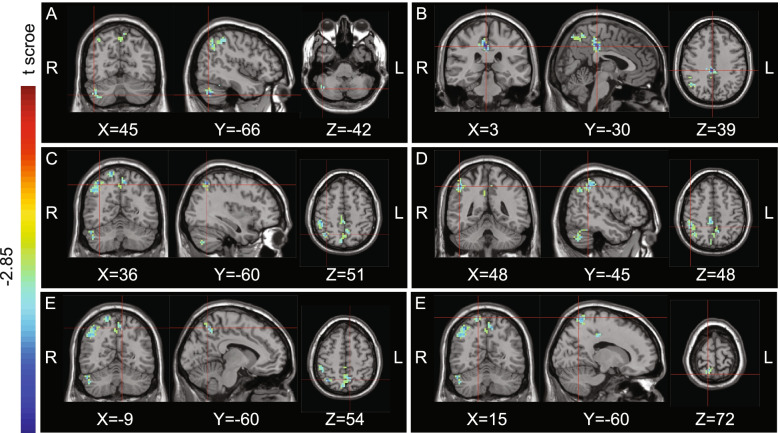
Regions showing significant differences in fALFF between R-CH patients during the non-painful period and normal controls, with a threshold of *P* < 0.01. The color bar indicates the *t*-score of brain regions with fALFF differences between CH patients during the non-painful period and normal controls, R: right; L: left. **A** The right cerebellum (x = 45, y = − 66, z = − 42). **B** The right cingulate gyrus (x = 3, y = − 30, z = 39). **C** The right superior parietal lobule (x = 36, y = − 60 z = 51). **D** The right inferior parietal lobule (x = 48, y = − 45, z = 48). **E** The right postcentral gyrus (x = − 9, y = − 60, z = 54). **F** The left precuneus (x = 15, y = − 60, z = 72)

fALFF values of the left cerebellum, left lentiform nucleus, left frontal lobe, left anterior cingulate, and right postcentral gyrus were significantly lower in the left headache than NC group (Fig. 1), while fALFF values of the right cerebellum, right cingulate gyrus, right superior parietal lobule, right inferior parietal lobule, right postcentral gyrus, and left precuneus were significantly lower in the right headache than NC group (Fig. 2) (*P* < 0.01). No fALFF value in the left CH or right CH groups was higher than the corresponding value in the NC group (Tables 1 and 2).

**Table 1:** The fALFF brain area was decreased in the left CH compared to the normal control

Brain regions	Hemisphere	BA	No. of voxels	Talairach coordinates (mm)			T-score (peak)
				x	y	z	
Left cerebellum	Left	–	17	−27	−51	−33	−3.473
Lentiform nucleus	Left	–	47	−24	3	−6	−4.4634
Frontal lobe	Left	10	29	−24	33	6	−3.6416
Anterior cingulate	Left	24	44	−18	36	18	−4.3421
Postcentral_R (aal)	Right	3	16	27	−33	51	−4.5212

Brain regions with greater fALFF changes in left CH patients during the non-painful period vs. normal controls. Threshold: *P* < 0.01; *fALFF* fractional amplitude of low-frequency Fluctuation, *CH* cluster headache, *BA* Brodmann’s area

**Table 2:** The fALFF brain area was decreased in the right CH compared to the normal control

Brain regions	Hemisphere	BA	NO. of voxels	Talairach coordinates (mm)			T-score (peak)
				x	y	z	
Right cerebellum	Right	–	68	45	−66	−42	−4.7791
Cingulate gyrus	Right	31	109	3	−30	39	−5.6906
Superior parietal lobule	Right	7	66	36	−60	51	−4.7168
Inferior parietal lobule	Right	40	81	48	−45	48	− 4.697
Precuneus_L (aal)	Left	7	96	−9	−60	54	−4.3352
Postcentral gyrus	Right	3	54	15	−60	72	−4.4526

Brain regions with greater fALFF changes in right CH patients during non-painful period vs. normal controls. Threshold: *P* < 0.01, *fALFF* fractional amplitude of low-frequency Fluctuation, *CH* cluster headache, *BA* Brodmann’s area

### Correlation analysis of clinical characteristics and mean fALFF values

**Fig. 3:**
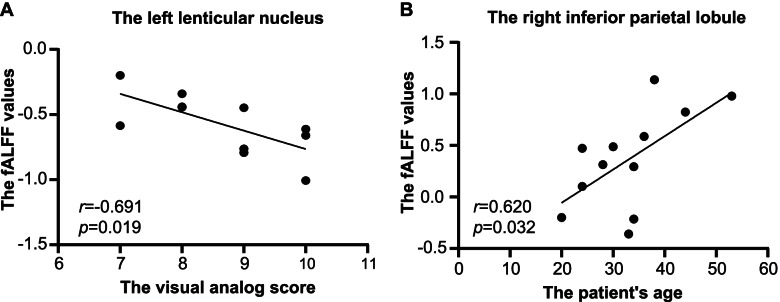
Correlation analysis of clinical characteristics and mean fALFF values. The fALFF value of the left lenticular nucleus was negatively correlated with the visual analog scale score (**A**) in the L-CH group, but that of the right inferior parietal lobule positively correlated with patient age (**B**) in the R-CH group

A negative correlation was observed between the left lenticular nucleus fALFF values and VAS score in the left CH group (*r* = − 0.691, *P* = 0.019) and the right inferior parietal lobule fALFF values were positively correlated with patient age (*r* = 0.620, *P* = 0.032). (Fig. 3). Other regional fALFF values and clinical characteristics were not correlated (Supplementary Table 4 and 5).

## Discussion

To the best of our knowledge, this is the first RS-fMRI investigation of patients with CH to use fALFF values. We attempted to determine alterations in intrinsic brain activity and to explore their relationships with clinical parameters using fALFF method that took into consideration the spatial distribution and amplitude of spontaneous LFOs. We found a decrease in fALFF values in certain brain areas, such as the cerebellum, lentiform nucleus, frontal lobe, and right postcentral gyrus, indicating abnormal activity in multiple intracranial brain regions during the non-painful state in a cluster period of CH patients. This suggests that the pain related brain network of CH patients in a cluster period is affected even in non-painful state.

Because CH attacks are usually unilateral, we divided the patients into left and right headache groups. Patients in the left headache group showed only left headache during the attack, while only right pain was observed in the right group. The abnormally active brain areas were not the same between the left and right headache groups. This may be due to differences in structure [20] and function [21] between the bilateral cerebral hemispheres. However, fMRI research has demonstrated that cerebral asymmetries are never absolute; even for strongly left-lateralized functions such as language, the right hemisphere makes a significant contribution [22]. Second, brain network functions in bilateral hemispheres are not completely symmetrical. Imaging, clinical, and behavioral data demonstrate hemispheric asymmetries in attentional networks, as revealed by the lateralized attention network test [23]. The orienting and alerting networks show left hemisphere dominance [24], while the executive control network is right hemisphere-dominant [25]. Therefore, we believe that studies of CH should group participants according to headache side.

Based on regional homogeneity, we previously showed that activity in brain regions such as the bilateral middle prefrontal cortex, right posterior cingulate cortex, and left dorsolateral prefrontal cortex decreases during a cluster period [26]. Another study reported greater hypometabolism in the perigenual anterior cingulate, prefrontal and orbitofrontal cortices, as revealed by positron emission tomography, in CH patients compared to healthy volunteers [27], which supported our results. Notably, the cingulate gyrus, superior parietal lobule, inferior parietal lobule, precuneus, and frontal lobe constitute the hub region of the DMN, which is the most studied brain network, was shown to be active in the resting state (i.e., in the absence of thinking), but was dormant in the task state [28]. In our study, activity of the default network in CH patients decreased rather than increased, suggesting that the activity of the DMN in CH patients is reduced on the same side as the headache during the resting state.

This study showed that both left- and right-sided CH were associated with decreased activity in the ipsilateral cerebellum. Although the specific mechanism underlying the involvement of the cerebellum in CH remains unclear, a previous structural and functional imaging study suggested that the cerebellum might be involved in the pathogenesis of CH [29–31]. Morelli et al. were the first to study brain activation patterns in patients with episodic CH using RS-fMRI, and reported that the cerebellum, as an “unconventional” pain-related brain area, was abnormally activated [29]. Farago et al. used fMRI to study brain activation patterns and intensity in CH patients during the non-painful period. They also found that the ipsilateral cerebellum and cerebellar network (CEN) of headache patients were functionally connected, i.e., were activated simultaneously [32]. However, in this study, a decrease in cerebellar activation on the side ipsilateral to the pain was seen. There are several possible explanations for the abnormalities observed in our patients. First, we speculate that this may have been due to the research methods used. In the former study, independent component analysis of functional integration was performed, while we used fALFF for functional differentiation. In addition, the acquisition range of the low-frequency blood oxygen signal was different: a “low-frequency” blood sample was collected (0.02–0.01 Hz) in the former study, while in this study the frequency range was 0.01–0.08 Hz. Furthermore, cerebellar activity occurs during the presence of acute and chronic pain [33], and the occurrence of headache in patients with CH is a dynamic process. We speculate that cerebellar activity is also dynamically changed.

The fALFF values in the right postcentral gyrus decreased in both of our groups. The central posterior gyrus is in the primary somatosensory area and somatosensory network, which is mainly involved in the localization and recognition of pain, and receives signals from the thalamus [34]. Both the current study and a previous one observed that postcentral gyrus activity decreased in chronic pain, which may be related to the suppression of pain perception under long-term stimulation [35].

The activity of the left lenticular nucleus decreased in our left CH headache group. In addition, a negative correlation was observed between the fALFF value in the left lenticular nucleus and VAS score; the lower the fALFF value, the higher the VAS score, so the more severe the pain. A study that utilized voxel-based morphometry to evaluate abnormal patterns of local gray and white matter in patients with CH reported that the volume of the lenticular nucleus was decreased in patients with CH [36]. Previous studies on the subcortical microstructure of right and left CH patients reported “higher” diffusion parameters of the lenticular nucleus [37], indicating microstructural disintegration and atrophy. The basal ganglia and subcortical structures have been proposed to play a central role in nociception. Furthermore, basal ganglia structures are activated during the application of painful stimuli [38]. Also, reduced fractional anisotropy was found in the corpus callosum and some frontal and parietal white matter tracts in CH patients, mainly on the contralateral side of the pain [39]. We speculate that the fALFF value of the lenticular nucleus may be useful when choosing the CH treatment during the non-painful CH period.

The activity of the parietal lobe was decreased in our right CH group, including the right superior parietal lobule, right inferior parietal lobule, right postcentral gyrus, and left precuneus lobe. A brain network study reported that the functional connections between the parietal lobe, insular lobe, and cerebellum were significantly stronger in CH than migraine patients, in both RS-fMRI and task-state fMRI analyses [40]. A study based on voxel-based morphometry reported that the grey matter volume of the left inferior parietal lobule was decreased, while the grey matter volume of the right cuneus was increased in patients with CH [36].

Our findings have several potential implications for clinical treatment. Currently used analgesics, as well as oxygen, are not completely effective for CH. Given the hypofunctioning of the brain network on the side ipsilateral to the during period of CH, could non-invasive treatments be used? For example, meditation changes the activity of the DMN and improves the coupling of multiple brain networks by changing the functional connection between brain regions [41]. Transcranial magnetic stimulation (TMS) increases brain activity through single or repetitive pulses, which affect neuroplasticity and the functional connections among brain networks [42]. A naturalistic study reported that treating chronic CH with maintenance sessions of repetitive TMS (rTMS) of the motor cortex reduced the intensity of permanent and paroxysmal pain, as well as the daily number of painful attacks. However, there are few reports on the use of rTMS to treat CH [43]. Our results may lead to less-invasive treatments for CH.

Our study had several limitations. First, the sample size was small. The rarity of CH makes it difficult to conduct large, well-controlled studies. Second, a correlation analysis between clinical characteristics and fALFF changes in multiple brain areas should be evaluated cautiously, as multiple testing may lead to spurious significances. Third, our study was only concerned with the non-painful period of CH, as dynamic changes in fALFF during the onset of CH cannot be observed. Thus, a longitudinal follow-up study may be necessary; more evidence is needed to confirm that the changes in spontaneous brain activity observed herein were the result of CH.

## Conclusion

Our results provide new insight into the pathogenesis of CH. Although the pathogenesis of CH is highly complex, dysfunction in multiple brain areas were involved in the non-painful CH period. It is necessary to study CH according to the headache side, given the lateralization of brain function.

## Supplementary Information

**Additional file 1.**

**Additional file 2: Supplementary Table 1.** The general characteristics of the cluster headache (CH) and control groups. **Supplementary Table 2.** Characteristics of cluster headache (CH). **Supplementary Table 3.** Accompanying symptoms of cluster headache (CH). **Supplementary Table 4.** The Correlations between clinical characteristics and fALFF values of abnormal brain regions in left CH patients. **Supplementary Table 5.** The Correlations between clinical characteristics and fALFF values of abnormal brain regions in right CH patients.

## Data Availability

The datasets used and/or analysed during the current study are available from the corresponding author on reasonable request.

## Abbreviations

CH: Cluster headache
fALFF: Fractional amplitude of low-frequency fluctuation
RS-FMRI: Resting-state functional magnetic resonance imaging
NC: The normal Controls
DMN: Default-mode network
VAS: Visual analog scale
TMS: Transcranial magnetic stimulation
BA: Brodmann’s area
