# Supplementary material for: Brain regions involved in fractional amplitude of low-frequency fluctuation in cluster headache patients: a resting-state functional MRI study
Source: BMC Neurol. 2022 Sep 7;22:336. doi: 10.1186/s12883-022-02863-3 (PMC9450424; doi:10.1186/s12883-022-02863-3)
Supplement: Supplementary file 2 — Additional file 2: Supplementary Table 1. The general characteristics of the cluster headache (CH) and control groups. Supplementary Table 2. Characteristics of cluster headache (CH). Supplementary Table 3. Accompanying symptoms of cluster headache (CH). Supplementary Table 4. The Correlations between clinical characteristics and fALFF values of abnormal brain regions in left CH patients. Supplementary Table 5. The Correlations between clinical characteristics and fALFF values of abnormal brain regions in right CH patients. [file 12883_2022_2863_MOESM2_ESM.docx]

Supplementary table 1. The general characteristics of the cluster headache (CH) and control groups

| **Characteristic** |  | **CH group** | **Control** | ***P* value** |
| --- | --- | --- | --- | --- |
| Age (years) | Mean ± SD | 33.5 ± 10.8 | 33.8 ±10.9 | 0.892 |
|  | Onset age | 25.0 ± 8.7 | NA |  |
| Sex | Female | 5 | 5 | 1 |
|  | Male | 18 | 18 |  |

Supplementary table 2. Characteristics of cluster headache (CH)

| **Characteristic** | | **Localization of CH** | | ***P* value** |
| --- | --- | --- | --- | --- |
|  |  | **Left (n=11)** | **Right (n=12)** |  |
| Headache attack duration | ≤30 min | 1 (9.1%) | 2 (16.7%) | 1 |
|  | >30 min | 10 (90.9%) | 10 (83.3%) |  |
| Frequency of attacks | ≤1 time/day | 8 (72.7%) | 10 (83.3%) | 0.640 |
|  | >1 time/day | 3 (27.3%) | 2 (16.7%) |  |
| Cluster bout duration | ≤4 weeks | 5 (45.5%) | 7 (58.3%) | 0.537 |
|  | >4 weeks | 6 (54.5%) | 5 (41.7%) |  |
| Disease duration | ≤10 years | 7 (63.6%) | 5 (41.7%) | 0.292 |
|  | >10 years | 4 (36.4%) | 7 (58.3%) |  |
| Visual analogue scale score |  | 8.64±1.12 | 9.08±1.31 | 0.935 |

Supplementary table 3. Accompanying symptoms of cluster headache (CH)

| **Characteristic** | | **Localization of CH** | | ***P* value** |
| --- | --- | --- | --- | --- |
|  |  | **Left (n=11)** | **Right (n=12)** |  |
| Conjunctive injection | Yes | 10 (90.9%) | 11 (91.7%) | 1 |
|  | No | 1 (9.1%) | 1 (8.3%) |  |
| Lacrimation | Yes | 9 (81.8%) | 12 (100.0%) | 0.217 |
|  | No | 2 (18.2%) | 0 (0.0) |  |
| Nasal congestion | Yes | 4 (36.4%) | 8 (66.7%) | 0.146 |
|  | No | 7 (63.6%) | 4 (33.3) |  |
| Rhinorrhea | Yes | 5 (45.5%) | 9 (75.0%) | 0.214 |
|  | No | 6 (54.5%) | 3 (25.0%) |  |
| Nausea | Yes | 8 (72.7%) | 6 (50.0%) | 0.400 |
|  | No | 3 (27.3%) | 6 (50.0%) |  |
| Vomiting | Yes | 6 (54.5%) | 3 (25.0%) | 0.214 |
|  | No | 5 (45.5%) | 9 (75.0%) |  |
| Photophobia | Yes | 5 (45.5%) | 3 (25.0%) | 0.400 |
|  | No | 6 (54.5%) | 9 (75.0%) |  |
| Phonophobia | Yes | 4 (36.4%) | 3 (25.0%) | 0.667 |
|  | No | 7 (63.6%) | 9 (75.0%) |  |

Supplementary table 4. The Correlations between clinical characteristics and fALFF values of abnormal brain regions in left CH patients.

|  | cerebellum | | lentiform nucleus | | frontal lobe | | anterior cingulate | | postcentral_R (aal) | |
| --- | --- | --- | --- | --- | --- | --- | --- | --- | --- | --- |
|  | *r* | *P* | *r* | *P* | *r* | *P* | *r* | *P* | *r* | *P* |
| Age | 0.22 | 0.949 | -0.356 | 0.283 | 0.031 | 0.927 | 0.308 | 0.356 | 0.191 | 0.574 |
| Headache attack duration | 0.18 | 0.58 | 0.478 | 0.137 | 0.03 | 0.93 | 0.219 | 0.518 | 0.159 | 0.641 |
| Frequency of attacks | 0.178 | 0.601 | 0.585 | 0.059 | -0.8 | 0.814 | 0.453 | 0.162 | 0.013 | 0.97 |
| cluster bout duration | -0.457 | 0.158 | 0.188 | 0.579 | -0.215 | 0.526 | 0.231 | 0.495 | 0.067 | 0.845 |
| Disease duration | 0.227 | 0.501 | -0.392 | 0.234 | -0.227 | 0.504 | 0.149 | 0.662 | 0.096 | 0.778 |
| Visual analogue scale score | -0.187 | 0.583 | **-0.691** | **0.019** | -0.526 | 0.097 | -0.311 | 0.352 | -0.058 | 0.866 |

Supplementary table 5. The Correlations between clinical characteristics and fALFF values of abnormal brain regions in right CH patients.

|  | Cerebellum | | Cingulate gyrus | | Superior parietal lobule | | Inferior parietal lobule | | Precuneus_L | | Postcentral gyrus | |
| --- | --- | --- | --- | --- | --- | --- | --- | --- | --- | --- | --- | --- |
|  | *r* | *P* | *r* | *P* | *r* | *P* | *r* | *P* | *r* | *P* | *r* | *P* |
| Age | 0.183 | 0.57 | 0.118 | 0.715 | -0.231 | 0.469 | **0.62** | **0.032** | 0.35 | 0.264 | -0.378 | 0.225 |
| Headache attack duration | -0.108 | 0.739 | -0.291 | 0.359 | 0.287 | 0.365 | -0.222 | 0.489 | -0.346 | 0.27 | 0.099 | 0.759 |
| Frequency of attacks | -0.005 | 0.988 | 0.05 | 0.869 | -0.053 | 0.074 | 0.281 | 0.377 | 0.368 | 0.239 | -0.227 | 0.479 |
| cluster bout duration | -0.345 | 0.272 | -0.414 | 0.181 | 0.161 | 0.616 | 0.041 | 0.9 | -0.195 | 0.545 | 0.373 | 0.233 |
| Disease duration | 0.116 | 0.72 | 0.236 | 0.46 | -0.566 | 0.055 | 0.389 | 0.211 | 0.565 | 0.056 | 0.076 | 0.814 |
| Visual analogue scale score | -0.159 | 0.623 | 0.434 | 0.159 | -0.137 | 0.671 | 0.253 | 0.428 | 0.323 | 0.305 | 0.01 | 0.754 |
